# Cyanidin-3-o-Glucoside Pharmacologically Inhibits Tumorigenesis via Estrogen Receptor β in Melanoma Mice

**DOI:** 10.3389/fonc.2019.01110

**Published:** 2019-10-22

**Authors:** Mei Liu, Yaqi Du, Haiwen Li, Li Wang, Donata Ponikwicka-Tyszko, Weronika Lebiedzinska, Agata Pilaszewicz-Puza, Huijiao Liu, Lijun Zhou, Hanlu Fan, Mingming Wang, Hua You, Slawomir Wolczynnski, Nafis Rahman, Yang-Dong Guo, Xiangdong Li

**Affiliations:** ^1^Beijing Advanced Innovation Center for Food Nutrition and Human Health, China Agricultural University, Beijing, China; ^2^State Key Laboratory of the Agro-Biotechnology, College of Horticultural Science, China Agricultural University, Beijing, China; ^3^Department of Pathology, Chinese PLA General Hospital, Beijing, China; ^4^Department of Biology and Pathology of Human Reproduction, Institute of Animal Reproduction and Food Research, Polish Academy of Sciences, Olsztyn, Poland; ^5^Department of Reproduction and Gynecological Endocrinology, Medical University of Bialystok, Bialystok, Poland; ^6^Department of Medical Pathomorphology, Medical University of Bialystok, Bialystok, Poland; ^7^Affiliated Cancer Hospital & Institute of Guangzhou Medical University, Guangzhou, China; ^8^Institute of Biomedicine, University of Turku, Turku, Finland

**Keywords:** estrogen receptor, C3G, CCNB1, apoptosis, melanoma

## Abstract

Expression patterns of estrogen receptors [ERα, ERβ, and G-protein associated ER (GPER)] in melanoma and skin may suggest their differential roles in carcinogenesis. Phytoestrogenic compound cyanidin-3-o-glucoside (C3G) has been shown to inhibit the growth and metastatic potential of melanoma, although the underlying molecular mechanism remains unclear. The aim of this study was to clarify the mechanism of action of C3G in melanoma *in vitro* and *in vivo*, as well as to characterize the functional expressions of ERs in melanoma. In normal skin or melanoma (*n* = 20/each), no ERα protein was detectable, whereas expression of ERβ was high in skin but weak focal or negative in melanoma; and finally high expression of GPER in all skin vs. 50% melanoma tissues (10/20) was found. These results correspond with our analysis of the melanoma survival rates (SRs) from Human Protein Atlas and The Cancer Genome Atlas GDC (362 patients), where low *ER*β expression in melanoma correlate with a poor relapse-free survival, and no correlations were observed between SRs and *ER*α or *GPER* expression in melanoma. Furthermore, we demonstrated that C3G treatment arrested the cell cycle at the G2/M phase by targeting cyclin B1 (CCNB1) and promoted apoptosis via ERβ in both mouse and human melanoma cell lines, and inhibited melanoma cell growth *in viv*o. Our study suggested that C3G elicits an agonistic effect toward ERβ signaling enhancement, which may serve as a potential novel therapeutic and preventive approach for melanoma.

## Introduction

Melanoma skin cancer with the rising incidence, originates from pigment-producing cells melanocytes, found in the basal layer of the epidermis and in the eye (1). Due to presence of the melanin pigment, melanoma is a disease that is accurately diagnosed earlier than most other malignancies and, thus, has been subjected to numerous therapeutic strategies. However, aside from early surgical resection, no therapeutic modality has enabled a high likelihood for a curative outcome (2).

Estrogen receptors (ERs), such as nuclear ERα and ERβ, and G protein-coupled estrogen receptor (GPER), are aberrantly expressed in a wide variety of malignancies other than estrogen-related cancers, and ERβ expression has been reported to decline in tumor tissues compared with normal tissues (3–6). A growing body of evidence shows important protective roles for ERβ in breast (3), melanoma (7), colorectal (6), prostate (5), and ovarian (4) carcinogenesis and their progression. Although the mechanism of this protective effect is unknown, the clinical association suggests that estrogen signaling is involved. Studies have shown that ERβ is the predominant ER subtype, while ERα is not detected in human melanoma cell lines (7) or melanocytic lesions (8, 9). Expression of ERβ expression has been suggested to be lower in melanoma tissues compared with the adjacent healthy skin (10), which could be a favorable prognosis factor of melanoma (10–12). Recently, an activation of GPER signaling that inhibits melanoma has been reported in two melanoma cell lines (13), which may expand the understandings of the melanoma carcinogenesis.

Studies have shown that consumption of fruit and vegetable rich diet may help to prevent melanoma (14). It is believed that phytochemicals, such as the widely distributed anthocyans (composed of anthocyanins and anthocyanidins) in fruits and vegetables induce cell apoptosis or cell cycle arrest in certain types of human cancers (15, 16), but exert little or no effect on the growth of normal cells (17, 18). Anthocyanins are a subgroup of flavonoids that are synthesized via the phenylpropanoid pathway, and are abundant in our daily diet, contributing to the intense color of many fruits, vegetables, and pigments (19). There are six particularly important anthocyanidins, including cyanidin, delphinidin, pelargonidin, malvidin, peonidin, and petunidin. Due to their instability in nature, the acylated anthocyanidins are most frequently forms and are glycosylated two or three-fold with monosaccharides (20). The anti-tumor effects of anthocyanins have been studied in melanoma (21), although the antitumor mechanism of individual anthocyanins remains elusive. The anthocyanin cyanidin-3-o-glucoside (C3G) has phytoestrogen activity by binding to ERs (22, 23), and possess higher selectivity for ERβ than for ERα. C3G is the most abundant anthocyanin pigment in many vegetables and fruits (24). The goal of this study was to reveal the underlying mechanistic effects of C3G in melanoma both *in vitro* and *in vivo*.

## Materials and Methods

### Compounds and Chemicals

C3G was purchased from Polyphenols AS Laboratories (Hanabryggene Technology Center, Norway). 17β-Estradiol (E2) and the ERs antagonist ICI 182,780, Dimethylsulfoxide (DMSO), phosphate buffered saline (PBS), and other chemicals were purchased from Sigma-Aldrich (Sigma, USA); ERα agonist PPT and ERβ agonist DPN were from Tocris Biosciences (Tocris Biosciences, UK).

### Antibodies

The monoclonal and polyclonal antibodies for cleaved-caspase-3 (#9664), caspase-3 (#9665), caspase-8 (#9746), and caspase-9 (#9508) were obtained from Cell Signaling Technology (CST, USA), RIP3 was obtained from Abcam (ab16090, Abcam, US); the polyclonal antibody for human ERβ (clone H-150, Santa Cruz Biotechnology, USA), the monoclonal antibody for human ERα (clone H222, Thermo Scientific, USA) were used as reported previously (7); the polyclonal antibody for mouse ERα (PA5-16476, Invitrogen Antibodies, USA), monoclonal antibody for mouse ERβ (NB200-305, Novus Biologicals, USA); the polyclonal antibody for human ERα (ABCA1866819, DAKO, Denmark); for immunohistochemical assay the monoclonal antibody for human ERβ (PPZ0506, R&D Systems, USA); for immunohistochemical assay the polyclonal antibody for human GPER (Sigma, HPA027052, USA); the polyclonal antibody for human/mouse GPER (Merck, SAB1304967, USA); human/mouse CCNB1 antibody (AF6000, R&D system, USA); monoclonal anti-rabbit for human TYR (1:100, Abcam, USA) polyclonal mouse anti-rabbit TYR (Cat No. 9319, Cell Signaling Technology, USA); (Monoclonal mouse anti-human Melan-A (Clone A103, DAKO; Denmark) were used. The monoclonal antibody for β-actin (sc-47778) was purchased (Santa Cruz Biotechnology, USA). Anti-mouse or anti-rabbit secondary horseradish peroxidase (HRP) conjugate was obtained (ZSGB-BIO co., China).

### Cell Cultures and Treatments

Cell lines for human breast cancer MCF7 (ATCC® HTB-22™) and MDA-MB-231 (ATCC® HTB-26™) and melanoma SK-MEL-1 (ATCC® HTB-67™) and A-375 (ATCC® CRL-1619™), human embryonic kidney 293 (ATCC® CRL-1573™), mouse B16-F10 (ATCC® CRL-6475™) were from National infrastructure of cell line recourse (NICLR, Beijing, China. NICLR is licensed by American Type Culture Collection, ATCC, USA). The B16-F10-luc cell line was purchased from Cold Spring Biotech co. (Beijing, China).

Cells were routinely maintained in Dulbecco's modified Eagle's medium (DMEM; Sigma, USA), supplemented with 10% FBS, 100 U/mL penicillin, and 50 U/mL streptomycin in a humidified atmosphere, containing 5% CO_2_ at 37°C.

MDA-MB-231 cells were maintained in L15 medium (Sigma, USA) supplemented with 10% FBS, 100 U/mL penicillin/50 U/mL streptomycin at 37°C. For all experiments, cells were used during the linear phase of growth.

### Cell Viability and Cytotoxicity Assay

Cells were suspended at a final concentration of 4 × 10^3^ cells/well in 96-well flat-bottomed microplates. Cells at 60–75% confluence were treated with C3G (50 and 150 μM) or vehicle DMSO alone for 48 h. C3G was dissolved in DMSO and the final concentration of DMSO stayed below 0.001% for all experiments. Cell morphology was observed via phase-contrast microscopy. For the cytotoxicity assay, measurement of cell membrane integrity was used as a parameter for cell death. Briefly, 2×CellTox Green Reagent (Promega, USA) was added to each well at the time of C3G dosing, and the provided lysis solution at 1:25 ratio was used as the positive control. Fluorescence was measured using a Tecan Infinite plate reader with 485–510 nm excitation and 520–530 nm emission. All experiments were performed in triplicate on three separate occasions and the data are presented as the mean of the respective triplicate.

### Cell Cycle Analysis

Cells were subjected to trypsin and the cells treated with DMSO or C3G were collected and washed in cold PBS, and gently fixed in 70% ethanol overnight at 4°C. After re-suspension in PBS, containing 0.01 mg/mL propidium iodide (PI; Sigma, USA) and 0.1 mg/mL RNase, cells were incubated in the dark for 30 min and stained cells were analyzed with a FC-500 flow cytometer (Beckman Coulter, USA). Cell cycle distribution was analyzed via MultiCycle software (Phoenix Flow Systems, USA). For each experiment, 10,000 cells were recorded. Each experiment was run in triplicate and carried out thrice.

### Apoptosis Assay for Cells

Cells were analyzed via Flow cytometric analysis: cells treated with DMSO or C3G were trypsinized, washed in cold PBS, dual stained with the AnnexinV/PI apoptosis detection kit (Sigma, USA) following the manufacturer's instructions, and signals were detected with FL-1 (FITC) and FL-3 (PI) detectors. Non-stained cells (lower-left quadrant) were considered live cells. Cells that stained with AnnexinV only (lower-right quadrant) were considered to be early apoptotic. Cells that stained with both AnnexinV and PI (upper-right quadrant) were considered to be late apoptotic or necrotic. Approximately, 5,000 cells were recorded per analysis.

Second, this method was performed as previously described (25): 4 micron tissue sections were deparaffinized in xylene and rehydrated through a series of decreasing ethanol concentrations. The slides were pretreated with hydrogen peroxide (3%) for 10 min to remove endogenous peroxidase, followed by antigen retrieval in a microwave in 10 mM citrate buffer (pH 6.0) for 15 min. The primary antibodies Cleaved Caspase-3 (9664s, Cell Signaling Technology, USA) were applied, followed by washing and incubation with the biotinylated secondary antibody for 30 min at room temperature. The slides were counterstained with hematoxylin and dehydrated in alcohol and xylene before mounting.

Apoptotic cell death also was confirmed using the terminal deoxynucleotidyl transferase-mediated dUTP nick end-labeling (TUNEL) technique as described by the *in situ* Cell Death Detection kit, POD (Roche, Germany) for DNA chromatin morphologic features used during quantification following the manufacturer's guidelines. For apoptosis quantification, the results were viewed under a fluorescence microscope (Olympus, Japan). Two observers counted at least 1,000 cells from more than 10 random microscopic fields.

### Terminal Deoxynucleotidyl Transferase-Mediated dUTP Nick End Labeling (TUNEL) Staining

TUNEL was performed to detect apoptosis in the melanoma tissue with the *in situ* cell death detection kit, POD (7seabiotech, China). Briefly, the samples were dewaxed through xylene and gradient ethanol. The 20 μl/ml of proteinase K was used to increase the sample permeability. After washing with PBS, the biotin-labeled reaction solution was added dropwise and incubated at 37°C for 1 h. After washing again, the pod reaction solution was added and the slides were incubated at 37°C for 30 min, Finally, DAB coloring solution was used for the color development.

### Chromatin Immunoprecipitation (ChIP) Assay

ChIP assays were performed according to the manufacturer's protocol (P2078, Beyotime Co., China) with slight modifications. Chromatin solutions were sonicated and incubated with anti-ERβ or with control IgG, and rotated overnight at 4°C. DNA-protein cross-links were reversed and chromatin DNA was purified and subjected to PCR analysis. The primer pair: 5′-CCGTAGAAATGGAAAGTGTGC-3′ and 5′-TGGAGAGCAGTGAAGCCAGT-3′ were used to amplify the predicated ERβ DNA interaction domain in CCNB1 promoter sequence. GAPDH was used as a negative control, the primer pair for GAPDH were: 5′-TACTAGCGGTTTTACGGGCG-3′ and 5′-TCGAACAGGAGGAGCAGAGAGCGA-3′. As IGF1 promoter region reported containing at least two sites for binding ERβ, IGF1 promoter was used as a positive control for the ERβ-DNA interaction, the primer pair were: 5′-CATAGTCTTTGCCTCATCGC-3′ and 5′-TTGTCCCAGTTGCCAAGT-3′. After amplification, PCR products were resolved on a 1.5% agarose gel and visualized by ethidium bromide staining.

### Measurement of Mitochondrial ROS

Cells treated with DMSO or C3G were removed from the culture medium at 24 h and stained with MitoTrackerRed CM-H2XRos (Invitrogen, USA) at 37°C in a humidified 5% CO_2_ atmosphere for 30 min. Cells were observed via laser scanning confocal microscope (Nikon, Japan).

### Isolation and Cultivation of Mouse and Human Primary Melanocytes

Mouse primary melanocytes were performed as previously described (26): punch skin biopsies were obtained from three C57BL/6C male mice (2-day old) on ice for anesthesia. First the underlying connective tissue was removed and digested in 0.2% dispase II at 4°C for 20 h. Then, epidermal tissue was separated from the underlying dermal tissue and digested in 0.25% trypsin and 0.02% EDTA at 37°C for 8 min. Finally, the dissociated cell suspensions were centrifuged. Total cell number and yield of viable cells were determined and maintained DMEM supplemented with 10% FBS, 100 U/mL penicillin and 50 U/mL streptomycin in a humidified atmosphere containing 5% CO_2_ at 37°C for all subsequent experiments.

The skin specimens were obtained from skin nevus in the Guangzhou Military Command, and informed consent was obtained from all patients. Briefly, the skin specimens were immersed in an iodine solution for 5 min, then washed extensively with cold saline. The subcutaneous tissue and dermis were removed, and the remaining skin was cut into small sections (0.5 mm thick) and placed in 0.25 % neutral protease overnight at 4°C to obtain the epidermis, which was then immersed in a solution of 0.25 % trypsin and 0.02 % EDTA at 37°C for 5 min. This digestion was terminated by the addition of serum. Single cell suspensions were obtained by pipette blowing, filtered through a 200 mesh filter for screening and centrifuged twice at 1,500 rpm for 6 min. M254 medium, supplemented with 1 % (v/v) human melanocyte growth supplement (HMGS2), 100 U/ml penicillin and 50 U/ml streptomycin, was added to the cells. The cells were then seeded into 25 cm^2^ culture flasks, at 5 × 10^5^ cells per flask, and cultured at 37°C in a humidified atmosphere.

### Validation of Purity of Melanocytes

Purity of melanocytes was determined by visual observation of cell morphology and histochemical analyses. Melanocytes were morphologically identified based on their characteristic dendritic morphology with multiple long processes and variable pigmentation. Tyrosinase (TYR) activity in melanocytes was assayed via L-DOPA reaction as previously described (27): culture media were removed and melanocytes rinsed twice in PBS, fixed for 20 min in fixative solution (ethanol:chloroform:acetic acid = 6:3:1), washed three times with PBS, and then incubated at 37°C for 18 h in the dark with 10 mM L-DOPA (Sigma, USA). Negative control melanocytes were incubated in the absence of L-DOPA. After incubation, the melanocytes were rinsed with distilled water, dehydrated, and mounted. Melanocytes that stained positive for TYR activity were observed via light microscopy.

### Plasmids

To experimentally determine whether ERβ bind to the promoter of CCNB1 the coding sequence of the *ER*β was cloned into the eukaryotic expression plasmid pEGFP-C1 using *XhoI* and *SalI* restriction sites. Meanwhile, the promoter sequence of *CCNB1* (from −1,000 bp to +100 bp) was amplified from the genome of Hela cell line IGR-39 (The human IGR-39 (BRAF V600E-mutant) and cloned into the luciferase reporter vector pGL3-basic (Promega, USA) using *KpnI* and *XhoI* restriction sites (pGL3- *CCNB1*). All generated constructs were confirmed by sequencing.

### Transient Transfection and Luciferase Activity Assay

Hela cells (ATCC® CCL-2™) were transiently transfected with reporter constructs together with expression vector using Lipofectamine 3000 (Invitrogen, USA) following the manufacturer's protocol. Briefly, the day before transfection, cells were seeded into the 96 well plate with normal growth medium and were 70–80% confluent at the time of transfection. The time of the started transfection was considered time 0. For reporter assays, cells were lysed 48 h after transfection and the supernatant was collected to measure the luciferase with the Dual-Luciferase Reporter Assay System (Promega, USA).

### RNA Extraction and Real-Time RT-PCR

Total RNA and quantitative real-time PCR (qPCR) were performed as previously described (28). Primer pairs are shown in Table 1. Data were analyzed with LightCycler® 480 software, Version 1.5 (Roche). Melting curves were run to confirm the specificity of the signal. Relative quantification of gene expression was performed using standard curves and normalized to the value for glyceraldehyde 3-phosphate dehydrogenase (GAPDH) in each sample. The films were scanned and quantified using NIH Image J 1.42 (http://rsb.info.nih.gov/ij/download. html) and each experiment was run in triplicate.

**Table 1:** The sequences of oligonucleotide primers and amplified products of real-time PCR.

**Gene name**	**Sequence of primer (5**′**-3**′**)**	**Amplified product (bp)**
GAPDH	F: TGTGTCCGTCGTGGATCTGA	149
	R: TTGCTGTTGAAGTCGCAGGAG	
Cyclin D2	F: AAGCCTGCCAGGAGCAAA	77
	R: ATCCGGCGTTATGCTGCTCT	
Cyclin B1	F: TGGCCTCACAAAGCACATGA	77
	R: GCTGTGCCAGCGTGCTAATC	
Cyclin E2	F: TGCTGCCGCCTTATGTCATT	78
	R: TCCGAGATGTCATCCCATTCC	
CDK2	F: CTGCCATTCTCACCGTGTCC	79
	R: AGCTTGATGGACCCCTCTGC	
CDK4	F: GGCCTTTGAACATCCCAAT	78
	R: TCAGTTCGGGAAGTAGCACAG	
CDK6	F: TGCGAGTGCAGACCAGTGG	150
	R: AGGTCTCCAGGTGCCTCAGC	
Cyclin A1	F:AATTGTGCCTTGCCTGAGTGA	133
	R:AAGAACTGCAGGTGGCTCCAT	

*F, forward; R, reverse; GAPDH, glyceraldehyde 3-phosphate dehydrogenase; CDK2, cyclin-dependent kinase 2; CDK4, cyclin-dependent kinase 4; CDK6, cyclin-dependent kinase 6*.

### Western Blot

Western blot analysis was performed as previously described (25). Protein was extracted from cell samples using the RIPA method and quantified via a Bio-Rad protein assay kit (cat. 500-0002; BioRad, USA). Protein samples were separated via SDS-PAGE and then transferred to a PVDF membrane (cat. IPVH00010; Millipore, USA). The membrane was incubated overnight at 4°C with the primary antibody diluted in 5% non-fat dry milk. The membranes were washed thrice and incubated for 45 min at room temperature with the HRP conjugated secondary antibody (Beijing, China). Each reaction was performed in triplicate in three independent Western blotting assays. The films were scanned and quantified using NIH Image J 1.42 (http://rsb.info.nih.gov/ij/download.html).

### *In vivo* Tumor Growth and Bioluminescent Imaging

This study was carried out in accordance with the principles of the Basel Declaration and recommendations of the Institutional Animal Care and Use Committee guidelines of China Agricultural University (CAU) under the permission number of AW02129102-3, the Institutional Animal Care and Use Committee of China Agricultural University (CAU). The protocol was approved by the Institutional Animal Care and Use Committee of China Agricultural University (CAU).

Four-week-old intact male C57BL/6 mice were purchased (Animal Center, Academy of Military Medical Sciences, China) and randomly divided into three groups (*n* = 10 for per group). The control and tumor model groups received a normal diet (ND; *n* = 10), while the C3G group was fed ND containing C3G 420 mg/kg (*n* = 10). At 6 weeks of age, mice were injected s.c. with B16-F10-luc cells (2 × 10^3^) in matrigel (BD Biosciences, USA). Body weights and food intake were monitored weekly. Tumor growth was monitored in real time with bioluminescent imaging of luciferase activity in live mice using the cryogenically cooled IVIS-imaging system (Calipers, USA). Tumor size was measured weekly using calipers, and the volume was calculated with the formula [(4/3π${\text{r}}_{1}^{2}$ × r_2_) (0.125)], where r_1_ was the smaller radius and r_2_ was the larger radius. At 9 weeks of age, mice were sacrificed. Four weeks post injection, mice were euthanized and tumors were removed, weighed, and identified via hematoxylin and eosin staining and cleaved-caspase-3 histological staining.

### Human Samples of Melanoma

Twenty cases of melanoma were retrospectively reviewed for their histopathological features; patients were seen at the Chinese PLA General Hospital in Beijing, China, in 2012–2013. The study was approved by the Ethics Committee of the Chinese PLA General Hospital.

### Gene Expression Correlation Analysis and Overall Survival Analysis

The TCGA SKCM dataset (*n* = 352) were download in FPKM (Fragments per kilo-base of exon per million reads mapped) format and transferred to TPM (Transcripts per million reads) format. Gene expression correlation and scatter plot were analyzed by R program. Overall survival analysis was analyzed using the R package “survival.”

### The Quantification of Microvasculature Density

Microvessel density (MVD) was quantified according to our previous study (25): slides were scanned at low power (×100) to identify the “hot spots” the areas of highest neovascularization. The average vessel count for the five “hot spots” was calculated as MVD. The results were calculated as MVD/mm^2^ (vessels/mm^2^). Differences were deemed significant with *p* < 0.01 and *n* = 20 fields were compared.

### Immunohistochemistry

This method was performed as previously described (29): 4 micron tissue sections were deparaffinized in xylene and rehydrated through a series of decreasing ethanol concentrations. The slides were pretreated with hydrogen peroxide (3%) for 10 min to remove endogenous peroxidase, followed by antigen retrieval in a microwave in 10 mM citrate buffer (pH 6.0) for 15 min. The primary antibodies were applied, followed by washing and incubation with the biotinylated secondary antibody for 30 min at room temperature. The slides were counterstained with hematoxylin and dehydrated in alcohol and xylene before mounting.

### ERα and ERβ Transactivation Assays

To assess activation of both human ERα and ERβ, ERα and β Reporter Assay Systems utilize non-human mammalian cells were performed (Indigo Biosciences, USA). Briefly, compounds were diluted in a medium provided by the manufacturer. After warmed to 37°C, the cell recovery medium provided in the assay kit was added to the tube of frozen reporter cells. To allow the cells attached firmly, the cell suspension (100 μL) was dispensed into the wells of a 96-well assay plate and incubated for 4 h. The test compounds (100 μL) were added to the cells at the indicated concentrations and incubated for 24 h. Luciferase activity was measured the luciferase with the Dual-Luciferase Reporter Assay System (Promega, USA).

### Competitive Binding Assays

To assess the competitive binding assays, the PolarScreen ERα and ERβ Competitive Binding Assay Kits Green (Life Technologies, USA) were used according to the manufacturer's protocol. Recombinant human ERα (25 nM) and ERβ (23 nM) and 4.5 nM Fluormone ES2 Green (fluorescently labeled estradiol) were incubated for 2 h with the test compounds, respectively. Fluorescence polarization was quantified using a Flex Station 3 (Molecular Devices, USA). IC50 values (the ligand concentration that yields 50% inhibition of Fluormone ES2 Green) were determined from competitive binding curves generated using GraphPad Prism ver. 6.01 for Windows (GraphPad Software, USA).

### Statistical Analysis

One-way ANOVA and Dunnett's *post hoc* tests were performed for all statistical analyses using the SPSS18.0.1 Package (SPSS Inc., Chicago, IL). Differences with *p* < 0.05 *), *p* < 0.01 (**), or *p* < 0.001(***) were considered statistically significant. Values are presented as the mean ± SEM (standard error of the mean).

## Results

### Low ERβ Expression in Melanoma Patients Was Critical for the Survival Rate in Patients

**Figure 1:**
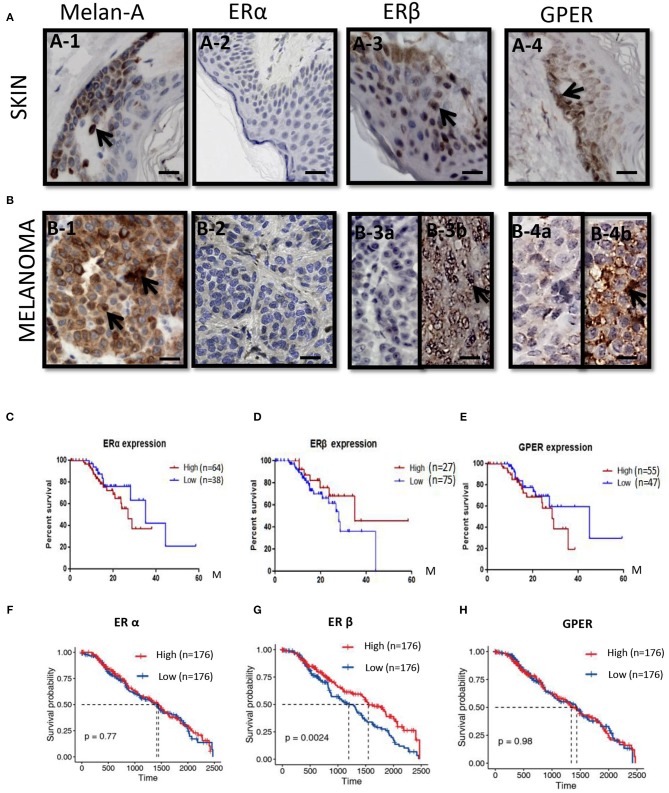
ERs expressions and the survival rates in melanoma patients. **(A)** Immunoshitochemical staining of Melanin (A–1), ERα (A–2), ERβ (A–3), and GPER (A–4) in skin (*upper panel*) (A–1–A–4) and **(B)** melanoma (*lower panel*) (B–1–B–4), where normal human skin serves as control. Arrows indicates the positive signals. Kaplan–Meier survival curves of the expression of *ER*α **(C,F)**, *ER*β **(D,G)**, and *GPER* **(E,H)** in all patients from the Human Protein Atlas **(C–E)** and TCGA **(F–H)**. Scale bar is 100 μm.

**Figure 2:**
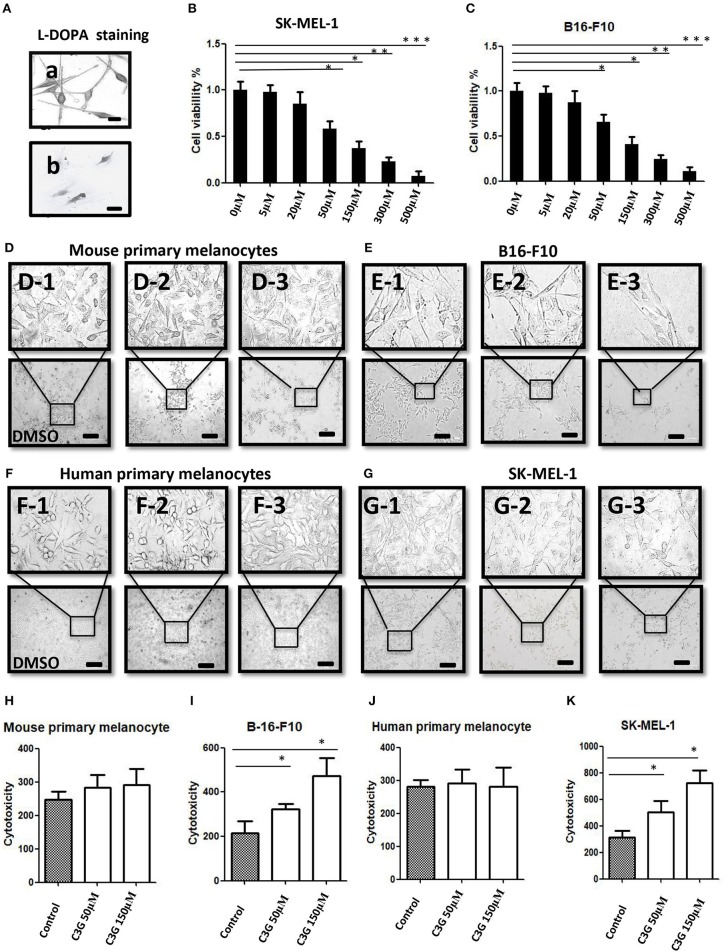
C3G inhibits growth of melanoma cells. **(A)** L-DOPA staining of cultured human and mouse primary melanocytes. **(B,C)** Cell viability was measured by treating the SK-MEL-1 **(B)** and B16-F10 **(C)** melanoma cells with different concentrations of C3G. DMSO served as the vehicle control. **(D–G)** Mouse (D1–3) and human (F1–3) primary melanocytes, as well as mouse melanoma B16-F10 (E1–3) cells and human SK-MEL-1 (G1–3) were treated without or with 50 and 150 μM C3G. DMSO served as the vehicle control, and cell morphology was observed by phase-contrast microscopy. Scale bar is 100 μm. **(H–K)** Cytotoxicity of doses (50 and 150 μM) of C3G in mouse primary melanocytes **(H)** and in mouse melanoma B16-F10 **(I)**, as well as in and human primary melanocytes **(J)** and human SK-MEL-1 melanoma cells **(K)**. The results represent the mean ± SEM from three independent experiments. Differences with *p < 0.05, **p < 0.01, or ***p < 0.001 were considered significant.

**Figure 3:**
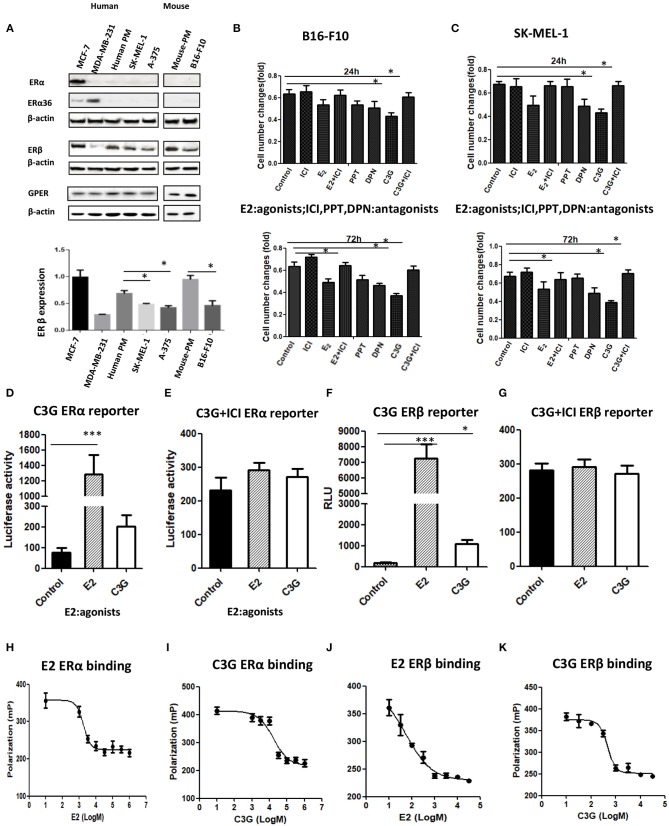
C3G binds to ERα and ERβ and possess ER transactivation activity. **(A)** Western blot analysis of ERα, ERβ, and GPER in human primary melanocytes and SK-MEL-1 and A-375 melanoma cells, and mouse primary melanocytes and B16-F10 melanoma cells. Human breast cancer cell lines MCF-7 and MDA-MB-231 were used as positive and negative controls for ERα, ERβ, and GPER, respectively. **(B,C)** Changes in cell numbers after treating the B16-F10 and SK-MEL-1 cells with E2 (10 nM), the ER antagonist ICI 182,780 (1.0 μM), the selective ERα agonist PPT, the selective ERα agonist DPN, and C3G, at 24 h (upper panel) and 72 h (lower panel), respectively. ERα reporter assay of cells treated with 50 μM C3G or 100 pM E2 in the absence **(D)** or presence **(E)** of 1.0 μM ICI 182,780. ERβ reporter assay of cells treated with 50 μM C3G or 100 pM E2 in the absence **(F)** or presence **(G)** of 1.0 μM ICI 182,780 for 24 h. RLU, relative light units. Data are mean ± SEM of three independent experiments. *p < 0.05 vs. control. Competitive binding curves for C3G displacement of fluorescein-labeled E2 from human ERα. ERα and fluorescein-labeled E2 were incubated for 2 h with a serial dilution of **(H)** E2, **(I)** C3G, in triplicate. IC50 corresponds to the concentration of test compound inhibiting binding of 4.5 nM Fluormore^TM^ ES2 Green to ERα by 50%. Competitive binding curves for C3G displacement of fluorescein-labeled E2 from human ERβ. ERβ and fluorescein-labeled E2 were incubated for 2 h with a serial dilution of **(J)** E2, **(K)** C3G, at least in triplicate. IC50 corresponds to the concentration of test compound inhibiting binding of 4.5 nM Fluormore^TM^ ES2 Green to ERβ by 50%. RBA values were calculated as 100 × IC50 (E2)/IC50 (test compounds). Inhibition by E2 was defined as 100%. Values are means ± SEM. Differences with *p < 0.05, **p < 0.01, or ***p < 0.001 were considered significant.

**Figure 4:**
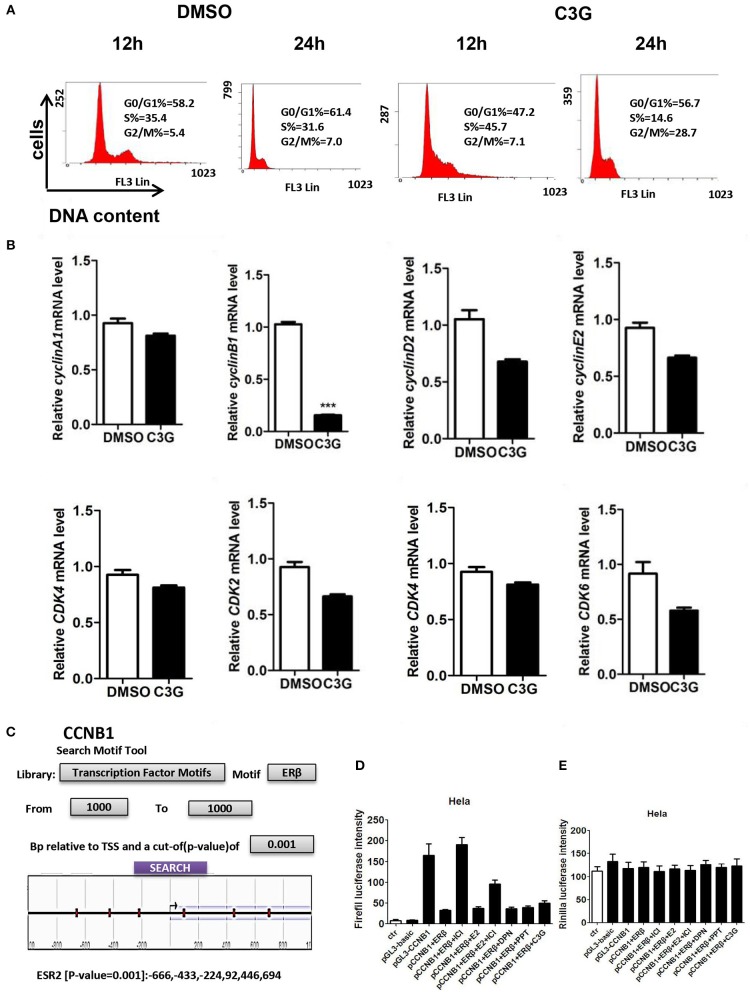
C3G treatment of B16-F10 cells and cell-cycle progression (arrested at G2/M phase). **(A)** C3G affects B16-F10 cell cycle progression measured via flow cytometry after 12 and 24 h treatments. DNA contents were measured via PI staining (x-axis) and the population of cells was measured (y-axis). **(B)** Relative mRNA expression levels of *cyclin B1, cyclin D1, cyclin E2*, and *CDK2, CDK4*, and *CDK6* following C3G 24 h treatment in B16-F10 cells. Data are mean ± SEM (*n* = 3). Triplicate measurements were performed for each experiment. Two-fold changes were used as the significant threshold. Differences with *p < 0.05, **p < 0.01, or ***p < 0.001 were considered significant. **(C)** The region of 1,000 nucleotides upstream of *CCNB1* was analyzed using the NCBI. Multiple predicted ERβ binding sites in the *CCNB1* promoter are marked in the sketch. **(D)** Luciferase activities were significantly activated by the pGL3-CCNB1 reporter vector and inhibited by DPN and C3G. **(E)** E is the background of luciferase. Differences with *p < 0.05, **p < 0.01, or ***p < 0.001 were considered significant.

To check the expressions of ERs in melanoma patients, immunohistochemical analyses were performed in melanoma paraffin sections. No ERα expression was observed in the normal skin (*n* = 10) and all melanoma samples (*n* = 20) (Figures 1A–2,B–2). Surprisingly, high ERβ expression was observed in the skin (Figure 1A–3), but was very weak positive (2/20, where high background could be observed) to negative in melanoma samples (18/20) (Figure 1B–3). High expression of GPER was observed in 50% (10/20) of the melanoma samples (Figure 1B–4), which consisted of focal negative (Figure 1B–4a) and focal positive (Figure 1B–4b) area, next to each other.

We analyzed with Kaplan–Meier plot for melanoma survival rates (SRs) and its correlation with ERα, ERβ, and GPER expression levels from the Human Protein Atlas. For the expression of *ER*α mRNA in melanoma patients (*n* = 75) across all melanoma subtypes, no significant difference in survival rate between the lower expression (*n* = 38) and the higher levels of *ER*α (*n* = 64) was observed (Figure 1C). Low expression of *ER*β mRNA in melanoma patients (*n* = 75) across all melanoma subtypes correlated with a poorer relapse-free survival compared with patients expressing high levels of *ER*β (*n* = 27) (Figure 1D). The 3-year survival rate for patients with higher *ER*β expression was 68%, whereas it was 37% for patients with lower *ER*β expression. The 5-year survival rate for patients with higher *ER*β expression was 45%, but the 5-year survival rate for patients with lower *ER*β expression was 0. High expression of *GPER* mRNA in melanoma patients (*n* = 55) across all melanoma subtypes correlated with a poorer relapse-free survival compared with patients expressing higher levels of *GPER* (*n* = 47) (Figure 1E). The 3-year survival rate for patients with higher *GPER* expression was 20%, whereas it was 65% for patients with lower *GPER* expression. In order to validate the expressions of these three ERs in the large numbers of the melanoma patients, we explored the expressions of *ER*α, *ER*β, and *GPER* in The Cancer Genome Atlas (TCGA) GDC Melanoma with 352 human samples. We confirmed that patients with high expression of *ER*β had better prognosis (*P* = 0.0024) (Figure 1G), but the survival rates of *ER*α or *GPER* with high or low expression did not show any significant changes (Figures 1F,H).

### C3G Inhibited the Growth of Mouse and Human Melanoma Cells

We previously demonstrated that C3G preferentially promoted apoptosis of triple negative breast cancer cells (TNBC) by directly binding to the ligand-binding domain (LBD) of ERα36 and could be a novel potential preventive/therapeutic agent against TNBC (25). In this study, we were interested in the role of C3G in melanoma, which is also an ER-related cancer (12). To analyze the treatment effects of C3G in melanoma, we isolated and cultivated mouse primary melanocytes from C57BL/6 skin tissue, and human primary melanocytes from the skin nevus. As TYR activity is a specific histochemical marker of melanocytes (30), L-DOPA staining was carried out to identify mouse and human primary melanocytes (Figure 2A). Based on the L-DOPA staining and the morphology of melanocytes with dendritic morphology, and variable pigmentation, the purity of the melanocytes was 93% for human and 97% for mouse.

The MTT assay was performed 48 h after treatment to determine the inhibitory concentration of the 50% (IC50) dose of C3G in the human and mouse melanoma cells (Figures 2B,C). We compared the cytotoxic effects of C3G in the SK-MEL-1 and B16-F10 cell lines. The results revealed that growths of the melanoma B16-F10 (Figures 2E,I) and SK-MEL-1 (Figures 2G,K) were inhibited by C3G treatment (50 and 150 μM) over 48 h compared to the DMSO vehicle control (Figures 2E,G,I,K). In contrast, the growth of primary melanocytes from mice and humans did not change significantly in either the 50 or 150 μM C3G treatments compared to the DMSO vehicle control (Figures 2D,F,H,J). These data demonstrate that C3G specifically inhibited the growth of B16-F10 and SK-MEL-1 melanoma cells.

### Decreased Expression of ERβ in Human and Mouse Melanoma Cell Lines

Growing evidence strongly suggests that ERβ plays a preventive role in the development and progression of melanoma (12). The expressions of ERα, ERβ, and GPER in human melanoma cell lines were analyzed by Western blot (Figure 3A). The MCF-7 and MDE-MB-231 breast cancer cell lines were used as the positive controls for ERα, ERβ, and ERα36. A band corresponding to ERα (66 kDa), a band corresponding to ERβ (59 kDa), and a band corresponding to ERα36 (36 kDa) at different expression levels were observed from the MCF-7 and MDE-MB-231 breast cancer cell lines, respectively (Figure 3A). No band corresponding to ERα or ERα 36 was detected in any of the primary melanocyte or melanoma cell lines analyzed (Figure 3A, lanes 1 and 2), confirming previous observations (11). A strong band and a very weak ERβ band were detected in the MCF-7 or MDE-MB-231 (positive and negative controls; lane 4), as shown previously (25). The human melanoma SK-MEL-1 and A-375 and mouse B16F10 cells lines (lane 4) expressed ERβ (59 kDa), but at lower levels (Figure 3A, lane 4) compared to the human and mouse primary melanocytes, respectively. Decreased expression of ERβ was observed in the human and mouse melanoma cells compared to primary melanocytes (Figure 3A, lane 4). Interestingly, no significant altered expression of GPER was observed in the human and mouse melanoma cells compared to primary melanocytes (Figure 3A, lane 6).

### ERβ Agonist DPN, but Not ERα Inhibited Melanoma Cell Proliferation

The selective ERβ agonist DPN decreased melanoma cell proliferation at a concentration of 10^−8^ M, while the selective ERα agonist PPT had no effect on melanoma cell proliferation at 10^−8^ M (Figures 3B,C). The natural estrogenic ligand E2 exerted some anti-proliferative effects on the melanoma B16F10 and A-375 cell lines at a concentration of 10^−8^ M (Figures 3B,C). C3G exerted a significant anti-proliferative effect on both melanoma cell lines at a concentration of 5 × 10^−5^ M. In a time course study, we observed the anti-proliferative activities of DPN, E2 (10^−8^ M), and C3G were completely counteracted by co-treatment of the cells with the ER antagonist ICI 182,780 (10^−6^ M) at 24 h (Figures 3B,C), and statistically significant results were obtained at 72 h (Figures 3B,C).

### C3G Binds to ERα and ERβ and Showed ER Transactivation Activity

C3G exhibited estrogenic activity both in human ERα and ERβ reporter assays at 50.0 μM (*p* < 0.05 and *p* < 0.01, respectively) (Figures 3D–G). C3G-mediated induction of estrogen response element-dependent luciferase activity was inhibited by co-treatment with 1 μM ICI 182,780 (Figures 3D,F), suggesting these effects were ERα- and ERβ-mediated, respectively. Our data suggest that C3G had phytoestrogenic activity mediated mainly via the ERβ signal.

To investigate whether the phytoestrogenic activity of C3G *in vitro* resulted from binding to ERα and ERβ, we calculated the approximate IC50 values using PolarScreen assays. E2 is positive control of C3G (Figures 3H,J). C3G exhibited the ability to bind to ERα and ERβ (Figures 3I,K). The IC50 values of E2 and C3G were 3.5 nM and 3.8 μM, respectively.

### C3G Decreases the *CCNB1* Expression Arrested Cell-Cycle at the G2/M Phase in B16-F10 Cells Directly via ERβ

We asked whether the B16-F10 cell death promoted by C3G was due to the cell-cycle intervention. DNA content of B16-F10 cells was assessed by flow cytometry to evaluate the effects of C3G on cell cycle progression. We observed a decrease in the S phase population after the 24 h C3G (150 μM) treatment, and the cells were arrested in the G2/M phase at 24 h (Figure 4A). The 24-h flow cytometric analysis of cells treated with C3G showed about a four-fold increase in the percentage of cells in the G_2_-M phase (28%) compared with cells not in that phase (7%; Figure 4A). Furthermore, we also tested mRNA expression of cell cycle regulatory molecules (*CCNB1, CCND1, CCNE2, CDK1* and *CDK2*, and *CDK6*) after 24 h of C3G (150 μM) treatment. We set a two-fold change as the threshold for significant expression of the genes, and observed a sharp decrease in *CCNB1* after the C3G (150 μM) treatment, while the other genes did not change significantly (Figure 4B). CCNB1 is known to be an ERβ response gene in breast cancer cells (31).

To determine whether C3G inhibited the expression of *CCNB1* via *ER*β, we analyzed the region of 1,000 nucleotides upstream of *CCNB1* using NCBI. We found multiple predicted ERβ binding sites in the *CCNB1* promoter (Figure 4C). To verify this prediction, we performed the luciferase assay. As illustrated in Figures 4D,E, luciferase was significantly activated by the pGL3-CCNB1 reporter vector and inhibited by the E2, PPT (ERα selective agonist), DPN (ERβ selective agonist), and C3G. ERα and ERβ antagonist ICI 182,780 induced pGL3-CCNB1 reporter vector luciferase activation was *ER*β dependent. The luciferase of control of pGL3-CCNB1 reporter vector was not affected by these molecules (Figure 4E). Taken together, the result indicated that *ER*β mediates the expression of *CCNB1* by binding to its promoter region. To determine whether CCNB1 is a direct target of ERβ, ChIP assay with the ERβ antibody in SK-MEL-1 cells was carried out. Data showed enrichment of both binding sites within the CCNB1 promoter region, indicating that the increases of mRNA and subsequent protein levels of *CCNB1* in melanoma cell lines are likely due to a direct interaction of ERβ with the *CCNB1* gene promoter (Figure S1A).

### Low ERβ and High *CCNB1* mRNA Expressions Correlate With Decreased Survival in Melanoma Patients

For evidence linking ERβ and CCNB1 expression to the progression of melanoma, immunohistochemical analyses were performed from the paraffin sectioned in human melanomas samples. By using the same paraffin sections for the expression of ERs, we detected strong CCNB1 expression in malignant melanoma tissues in 10/20 patients (50%) (Figure S1B), moderate expression in 5/20 patients (25%), and mild expression in 5/20 patients (25%). The bivariate correlation of *ER*β and *CCNB1* between these samples with Pearson's correlation value was −0.577 (*P* = 0.008). From Figures 1F–H, we noticed that high expression of *ER*β had better prognosis (*P* = 0.0024), but the survival rate of *ER*α or *GPER* with high expression and low expression did not shown any significant differences for the survival rate. Furthermore, we analyzed the correlation between *ER*α, *ER*β, or *GPER* and *CCNB*1, respectively. The expression of *CCNB1* was found to be negatively correlated with *ER*β (*R* = −0.259, *P* = 0.02292) (Figure S1D), but no correlations with the expressions of *ER*α and *GPER* were obtained (Figures S1C,E), which further confirmed our findings. These bioinformatical analyses further confirmed our immunohistochemical studies in human melanoma samples (shown in Figure 1).

### C3G Induced Melanoma Cells Death of via Apoptosis in the Caspase Cascade Pathway

**Figure 5:**
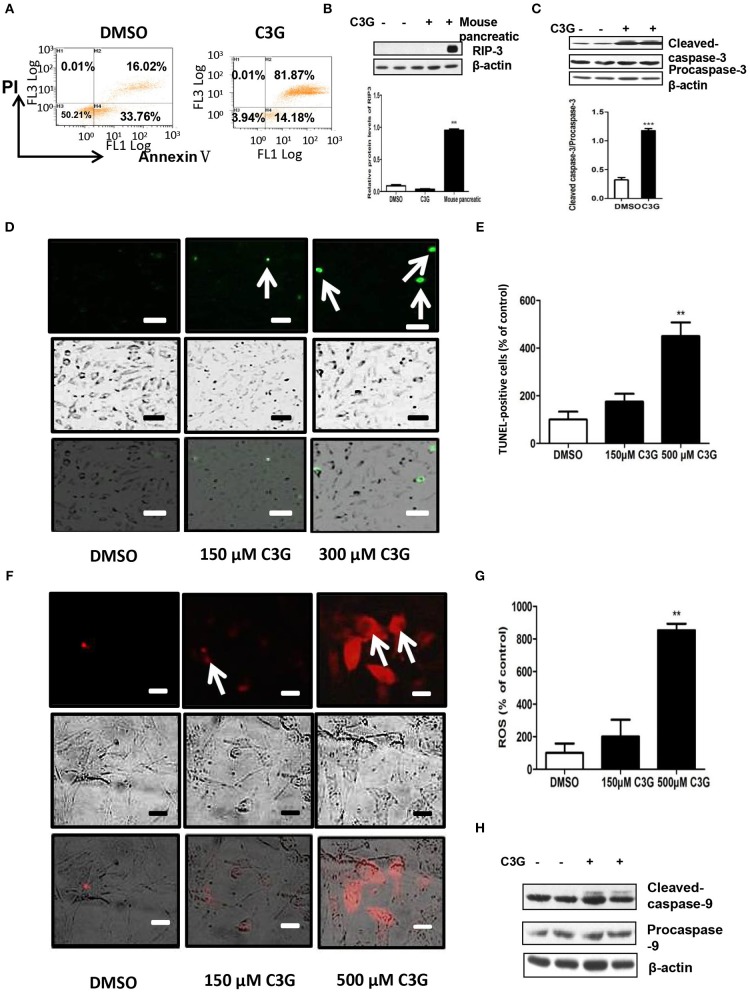
C3G promotes B16-F10 cells apoptosis via the mitochondrial pathway. **(A)** Annexin V/PI double staining of B16-F10 cells observed via flow cytometry after a 48 h C3G treatment. Phosphatidylserine was measured via Annexin V-FITC staining (X-axis), and apoptosis was measured via PI staining (Y-axis). **(B)** Upper panel: RIP3 expression in B16-F10 cells detected by Western blotting; the mouse pancreas was the positive control. Lower panel: relative RIP3 protein levels. **(C)** *Upper panel*: expression levels of cleaved-caspase-3 (17 and 19 kDa) and caspase-3 (35 kDa). β-actin was the internal control. The *lower panel* shows the relative protein levels of cleaved-caspase-3 in B16-F10 cells. **(D)** TUNEL staining to detect late apoptotic cells after C3G treatment. Late apoptotic cells have green nuclear regions (arrows), bar graphs show the number of TUNEL-positive cells. **(E)** The results represent mean ± SEM from three independent experiments. **(F)** Mitochondrial ROS fluorescent images were recorded with a fluorescence microscope 30 min after adding CM-H2XRos to the B16-F10 cells. DMSO cells remained unstained. **(G)** Bar graphs show the percentage of ROS-positive cells. **(H)** Western blot analysis of caspase 9 in C3G-treated mice tumors. The results represent mean ± SEM from three independent experiments. Differences with *p < 0.05, **p < 0.01, or ***p < 0.001 were considered significant.

We tested whether C3G promoted apoptotic cell death. Quantification by flow cytometry demonstrated that the C3G treatments (48 h) induced a dramatic increase in the amount of Annexin V/PI double-positive B16-F10 cells from 16.02 to 81.87% (Figure 5A). As both apoptosis (32) and necroptosis (33) are considered two of the most important mechanisms of cancer cell death, we determined which pathway was involved in C3G-induced cell death and cell death pathways. We first detected RIP3 expression in B16-F10 cells, using the mouse pancreas as a positive control (Figure 5B). We failed to detect RIP3 expression in B16-F10 cells treated with C3G, suggesting that necroptosis was not involved in C3G-induced cell death. Effector caspase-3 is cleaved-caspase-3, resulting in cleavage of critical cellular proteins and leading to cell death (34). We observed an increase in cleaved-caspase-3 subsequent to C3G treatment in B16-F10 cells (Figure 5C). TUNEL assay showed an increased number of dead cells after the C3G treatment (Figures 5D,E). These results indicated that C3G inhibited the growth of B16-F10 cells by promoting apoptotic death.

### C3G Induced Melanoma Cell Apoptosis via the Mitochondrial Pathway

We studied whether C3G induced apoptosis via mitochondrial dysfunction. Mitochondria plays an important role in the regulation of apoptosis by generating reactive oxygen species (ROS) and releasing cytochrome *c*, subsequently activating caspase-9 and caspase-3/7 (35–37). As shown in Figures 5F,G, C3G increased ROS in a dose-dependent manner in B16-F10 cells. In addition, we detected active caspase-9 (cleaved-caspase-9) expression in B16-F10 cells using Western blot analysis (Figure 5H). The C3G treatment promoted the expression of cleaved-caspase-9 compared to the DMSO control. These results indicate that C3G induces B16-F10 cell apoptosis via the mitochondrial pathway.

### C3G Inhibits the Growth of Mouse Melanoma Cells *in vivo*

**Figure 6:**
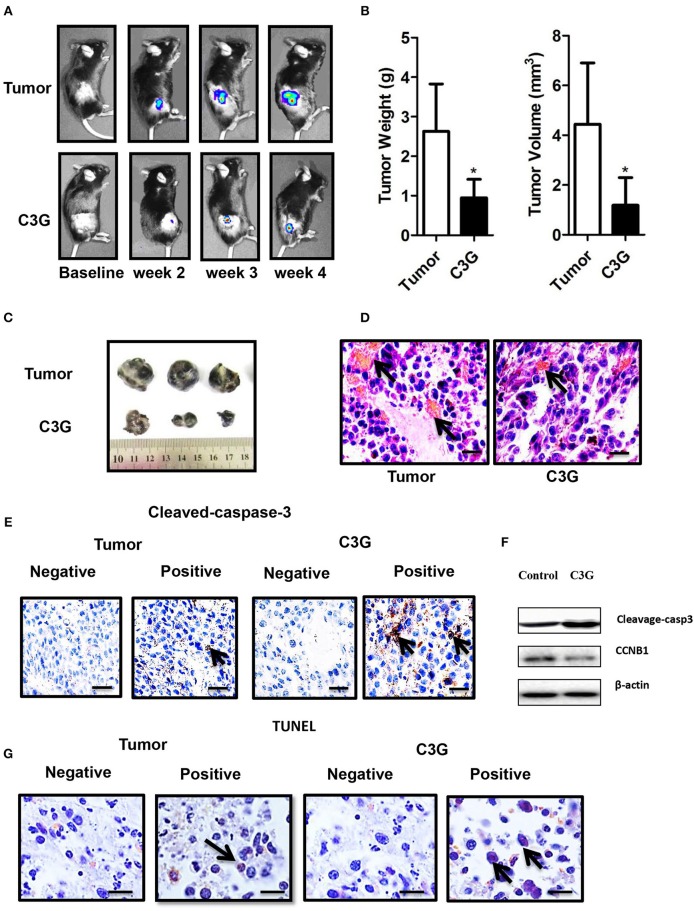
C3G inhibits the growth of B16-F10 cells *in vivo. In situ* tumor growth monitored via Xenogen IVIS imaging at different time points after implanting syngeneic B16-F10-luc tumors into male C57BL/6 mice (*n* = 10) either fed with control or C3G diet for 4 weeks. **(A)** Melanoma tumor growth was monitored in real-time via bioluminescent imaging of luciferase activity in live mice using the cryogenically cooled IVIS-imaging system from baseline to week 4 post implantation. **(B)** Graphical representation of tumor weight and tumor growth was monitored using Vernier calipers. **(C)** Photographic images of excised tumors were captured (*n* = 3). The largest tumor size in diameter is 2.0 cm. **(D)** Hematoxylin and eosin staining of tumor and capillaries are red (arrows). **(E)** Cleaved-caspase-3 was brownish in the cytoplasm (arrows) of the tumor and **(F)** Western blot analysis of caspase 3 and CCNB1 in C3G-treated mice tumors. **(G)**, TUNEL staining to detect late apoptotic cells of the tumor (left panel) and C3G-treated mice tumors (Right panel). Late apoptotic cells had brownish nuclear regions (arrows). Differences with *p < 0.05, **p < 0.01, or ***p < 0.001 were considered statistically significant. Scale bar is 100 μm.

The male C57BL/6 mice syngeneic graft model was used to examine the *in vivo* efficacy of C3G. We utilized stably expressing luciferase mouse melanoma (B16-F10-luc) cells to visualize and monitor melanoma growth in real-time. The results revealed that tumor growth and volume in mice treated with C3G were significantly smaller compared to that of control mice during the 4 weeks of observations (Figure 6A). Tumor size and weight further confirmed the inhibitory function of C3G when the mice were sacrificed (Figures 6B,C). A histological analysis showed that C3G treatment significantly inhibited micrangium formation (Figure 6D). The MVD was 12.00 ± 1.00/mm^2^ (tumor group) vs. 5.75 ± 0.95/mm^2^ (C3G group). Body weight and food intake were monitored weekly as indicators of overall health, and no differences were detected among the control, tumor, and C3G groups. Consistent with the *in vitro* results, apoptosis (cleaved-caspase-3 staining) and CCNB1 of the tumor specimens increased significantly, whereas CCNB1 of the tumor specimens decreased significantly in the C3G group (Figures 6E,F). The TUNEL assay also showed an increased number of dead cells in the mice tumor after C3G-treated (Figure 6G) These results illustrate that C3G also inhibits melanoma B16-F10 cells *in vivo*.

## Discussion

A growing body of evidence shows the preventive roles for estrogen and its ERs signaling pathway in melanoma, and suggests that ERs (nuclear ERs and GPER) may be the potential therapeutic targets associated with a suppressive function in them. By immunohistochemical analyses from melanoma patients (*n* = 20) and the mined data from the public databases, we demonstrated that ERβ expression was decreased in melanoma, and low expression of *ER*β in melanoma patients in all melanoma subtypes correlate with a poor relapse-free survival, whereas no correlations were observed between survival rates and the expression of *ER*α or *GPER* in melanoma. We also demonstrated that the C3G inhibits the growths in mouse and human melanoma cell lines via ERβ, and a mouse melanoma model. Our study suggested that agonistic effects of C3G targeted ERβ signaling enhancement, which could be a potential novel therapeutic and preventive approach for melanoma.

ERβ is believed to be one of the main prognostic factors in malignant melanoma (10), although the molecular role of ERβ in melanoma remains elusive. de Giorgi et al. study was the first one showing a statistical analysis that evaluated ERβ expression in malignant melanoma. Based on 66 malignant melanocytic lesions, they demonstrated that loss of ERβ may represent a crucial step in the development of malignant melanoma (10). Interestingly, Schmidt et al. (38) showed that ERβ is the predominant ER in melanocyte physiology. They also demonstrated that low ERβ expression occurs in progressively deeper malignant melanoma (*n* = 36). As for immunohistochemical assay, the results for human ERβ antibody are still contradictory and debated in last 20 years. Recently, Andersson et al. showed that the rarely used monoclonal antibody may specially target the human ERβ in immunohistochemistry (39). In accordance with de Giorgi's findings, by using this antibody, we also observed high expression of ERβ in human skin, and with very low or absent expression of the ERβ protein from 20 human melanoma samples.

By mining data from the Human Protein Atlas and TAGC, we found an inverse correlation between survival rate and the ERβ expression level in melanoma patients.

Marzagalli et al. demonstrated that ERβ (but not the ERα subtype) expression was decreased in most of the tested melanoma cell lines (7). Moreover, they reported that ERβ agonists exert antiproliferative activities in BLM melanoma cells by modulating cell cycle progressing factors (CCNB1, CCND3, and p27) and by blocking the G1-S transition phase without triggering the apoptosis pathway. They hypothesized that inhibition of these cell cycle-related proteins may not be directly related to ERβ, but to some of the ERβ downstream proteins. In line with their results, we also observed that ERβ (but not the ERα subtype) was expressed in most of the tested melanoma cell lines.

Natale et al. demonstrated that GPER was expressed in two of the tested melanoma cell lines (13). Moreover, they reported that activation of GPER signaling inhibits melanoma and improves response to immune checkpoint blockade, which extends the comprehensive mechanisms of estrogen signal action on the etiology and carcinogenesis in melanoma, and may provide the “proof of concept” for the new therapeutic strategy for melanoma. Surprisingly, we observed high GPER expression in 50% (10/20) of the melanoma samples, with their focal negative and positive area side by side, suggesting high heterozygosity of melanoma. Moreover, by mining the public data from Human Protein Atlas and TAGC, we demonstrated that low expression of *ER*β in melanoma patients across all melanoma subtypes correlate with a poor relapse-free survival, whereas no correlations were observed between survival rates and the expression of *ER*α or *GPER* in melanoma patients. To avoid any analysis bias, a large clinical investigation to valid the GPER as the biomarker for human melanoma is needed. Combining the studies from Natale et al. (13) and ours, it is highly likely that certain subtypes of melanoma might be more sensitive toward GPER therapy. As in Natale's study, systemically delivered GPER agonist was well-tolerated, and cooperated with immune checkpoint blockade in melanoma-bearing mice to dramatically extend survival rate in mice, with up to half of mice clearing their tumor. Whereas, for the clinical immune checkpoint blockade study, only 15–40% patients responded to PD-1/PDL-1 therapy (40, 41), which suggests GPER and immune checkpoint blockade therapies are tumor subtype-specific. ERβ has been known for its cell anti-proliferative effects (42). In this study, we showed that the luciferase activity of ERβ by E2 is seven-fold higher compared with C3G, but the cell proliferation by E2 did not significantly change compared to C3G treatment. The plausible explanation for this controversial result could be due to the differential ligand binding potentials to the nuclear receptor. It is well-known that ER ligands mediate their actions through recruiting of different co-activators or co-repressors by forming different multiprotein complexes on the basis of the shape of the ligand–receptor complex. These complexes influence the activity of the receptors, which activate or repress gene transcription for the cell proliferation (42). These above mentions issues could explain the differential activation results.

Anthocyanins have phytoestrogen activity via ERα and ERβ, and C3G has a higher binding affinity to ERβ than to ERα (22, 23). We also confirmed that C3G has a higher binding affinity to ERβ than ERα by the ERα and ERβ PolarScreen assay. The IC50 of C3G was ~1/1000 that of E2, indicating a weaker effect of the phytoestrogen compared to endogenous estrogen (22, 23). In this study, we demonstrated that the ER agonist C3G exerts its antiproliferative activity by inhibiting the expression of *CCNB1* and triggering the apoptosis pathway. Our data suggest that activation of C3G-induced ERβ may inhibit melanoma growth by blocking the G2-M transition phase. Specifically, we observed significantly reduced *CCNB1* expression at the transcriptional level in response to C3G, and that ERβ may be a favorable prognostic factor for melanoma (10–12). By using the ChIP assay with the ERβ antibody in melanoma cell, we also demonstrated that CCNB1 is a direct target of ERβ. We further demonstrated that Pearson's correlation coefficient between ERβ and CCNB1 was −0.577 (*P* = 0.008) from 20 paraffinized melanoma samples. One of the limitations of this study could be that the number of melanoma patient samples was small. A further large clinical investigation is needed to confirm this finding. CCNB1 is known to be an ERβ response gene in breast cancer cells (31). By mining the data from the Human Protein Atlas, we found an inverse correlation between survival rate and CCNB1 levels in melanoma patients. The newly opened TCGA provides a large amount of conveniently accessible primary tumor mutations, facilitating these conclusions regarding driver candidates. Furthermore, an inverse correlation was confirmed between expression levels of *ER*β and *CCNB1* in melanoma by mining data from the TCGA.

We observed that C3G treatment could inhibit the growth of melanoma cell lines, but not the primary melanocytes. So far a clear understanding of how ERβ exert its antiproliferative activity remains elusive. The plausible explanations for ERβ which elicits its proliferative and antiproliferative activities could be due the following reasons: (1) Numerous studies have demonstrated differentially expressed genes in the normal melanocytes vs. melanoma (43–47), Lu et al. has showed that tumor-specific promoters were specifically activated in melanoma compared to melanocytes (45). According to this study the potential candidates for the melanoma tumor specific promoters were Cox-2, CXCR4, EGP-2, and survivin [done by comparison analysis in melanoma cell lines, primary melanoma cells, and HEMs (a normal melanocyte control)]. These tumor-specific promoters may be the potential targets for C3G or drug targets for melanoma. Many studies have shown that compounds can inhibit the proliferation and differentiation of many different tumor cells (48, 49), including murine and human melanoma cells (50), and had limited or no effects on non-cancerous cell. This could also be the issue in our study for the melanoma-specific treatment effects with C3G. (2) It is well-known that ER ligands mediate their actions through recruiting of different co-activators or co-repressors by forming different multiprotein complexes on the basis of the shape of the ligand-receptor complex. This melanoma cell specific C3G treatment effects could have been also mediated through the specific nuclear receptors co-activator/corepressors complexes along with the basal transcriptional machinery in the context of the cells (42, 51).

So far there has been no report about the effects of C3G on melanoma in the existing literature. Rugina et al. have reported anthocyanins enriched extract (AEE) inhibited proliferation of metastatic melanoma B16-F10 cells in a concentration-dependent manner (21). They found 250 μg/mL AEE destroyed melanoma cell membrane integrity leading to apoptosis compared to the controls. Diaconeasa et al. have demonstrated the anti-proliferative effect of chokeberry and red grape anthocyanins rich extracts on melanoma (52, 53). Similar as what we observed in our study, anthocyanins from chokeberry and red grape have no negative influence on normal cells. In this study, we demonstrated that the anthocyanin C3G was an effective agent against melanoma both *in vitro* and *in vivo*. The preventive and therapeutic efficacy of C3G against melanoma needs to be further studied in a large clinical investigation. Our data reveal that C3G promoted melanoma cell apoptosis both *in vitro* and *in vivo* by binding to the ERβ, which, in turn, inhibited the expression of *CCNB1* and triggered the apoptosis pathway. Our results further indicate that C3G could be used as a chemopreventive or adjuvant treatment for melanoma.

## Data Availability Statement

All datasets generated for this study are included in the manuscript/Supplementary Files.

## Ethics Statement

This study was carried out in accordance with the principles of the Basel Declaration and recommendations of the Institutional Animal Care and Use Committee guidelines of China Agricultural University (CAU) under the permission number of AW02129102-3, the Institutional Animal Care and Use Committee of China Agricultural University (CAU). The protocol was approved by the Institutional Animal Care and Use Committee of China Agricultural University (CAU).

## Author Contributions

ML, YD, HLi, LW, LZ, HF, and MW performed the molecular and animal experiments. DP-T and WL performed the immunohistochemical studies from human samples. AP-P, HY, and SW were professional pathologists and carried out the histopathological analysis. HLiu was a bioinformatics and analysis the sequencing data from TCGA and PA databases. XL, YD-G, and NR designed the research, analyzed and interpreted the data for the work, and revising the manuscript.

### Conflict of Interest

The authors declare that the research was conducted in the absence of any commercial or financial relationships that could be construed as a potential conflict of interest.

## Supplementary Material

Figure S1 **(A)** ChIP assay was used to detect the direct binding of ERβ to the CCNB1 promoter. SK-MEL-1 cells were processed for ChIP using anti-ERβ antibody. CCNB1 represent prediction binding site. The IGF1 promoter primers were used as a positive control, and GAPDH primers were used as a negative control. **(B)** Immunoshitochemical stainings of Melanin A (left panel) from the normal human skin serve as the controls. Arrows indicated the positive signals. Immunoshitochemical stainings of CCNB1 (right panel) from the human melanoma samples. Gene expression correlation analysis for *CCNB1, ER*α*, ER*β, *GPER* in SKCM (TCGA dataset). The scatter plot shows Pearson correlation of *ER*α **(C)**, *ER*β **(D)**, and *GPER* **(E)** expression with expression of *CCNB1*.

## Abbreviations

C3G: cyanidin-3-o-glucoside (OR anthocyanin cyanidin-3-o-glucoside)
RIP3: receptor-interacting serine-threonine kinase 3
DMSO: dimethyl sulfoxide
ROS: reactive oxygen species.
