# Supplementary figures and images for: Cyanidin-3-o-Glucoside Pharmacologically Inhibits Tumorigenesis via Estrogen Receptor β in Melanoma Mice
Source: Front Oncol. 2019 Oct 22;9:1110. doi: 10.3389/fonc.2019.01110 (PMC6817467; doi:10.3389/fonc.2019.01110)

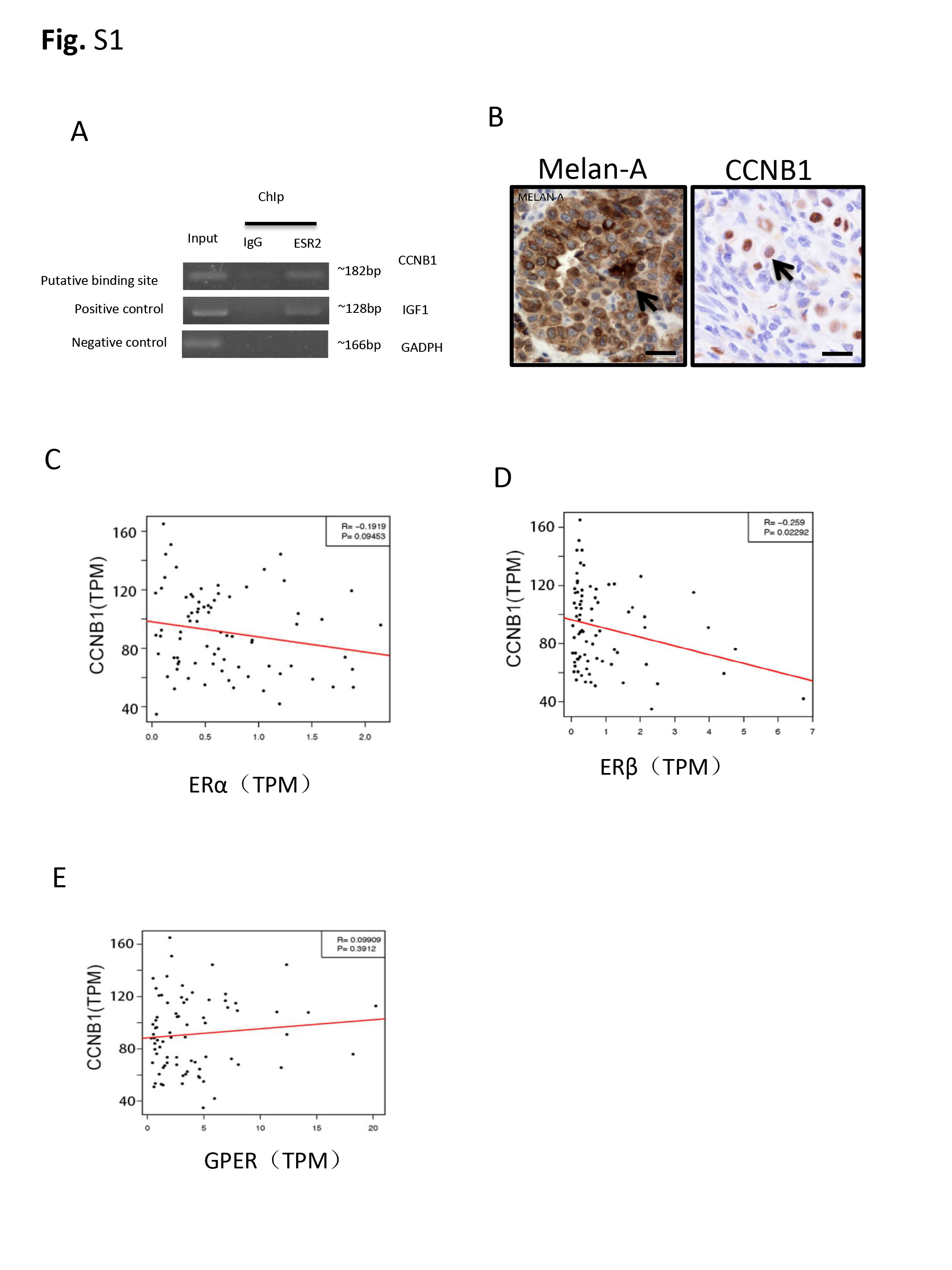

Supplement: Figure S1 — (A) ChIP assay was used to detect the direct binding of ERβ to the CCNB1 promoter. SK-MEL-1 cells were processed for ChIP using anti-ERβ antibody. CCNB1 represent prediction binding site. The IGF1 promoter primers were used as a positive control, and GAPDH primers were used as a negative control. (B) Immunoshitochemical stainings of Melanin A (left panel) from the normal human skin serve as the controls. Arrows indicated the positive signals. Immunoshitochemical stainings of CCNB1 (right panel) from the human melanoma samples. Gene expression correlation analysis for CCNB1, ERα, ERβ, GPER in SKCM (TCGA dataset). The scatter plot shows Pearson correlation of ERα (C), ERβ (D), and GPER (E) expression with expression of CCNB1. [file Image_1.jpg]
